# Spatial Memory Activity Distributions Indicate the Hippocampus Operates in a Continuous Manner

**DOI:** 10.3390/brainsci6030037

**Published:** 2016-08-26

**Authors:** Brittany M. Jeye, Jessica M. Karanian, Scott D. Slotnick

**Affiliations:** Department of Psychology, Boston College, Chestnut Hill, MA 02467, USA; jessica.karanian@bc.edu (J.M.K.); sd.slotnick@bc.edu (S.D.S.)

**Keywords:** recollection, source memory, fMRI, continuous model, unequal variance model, threshold model, receiver operating characteristic, ROC

## Abstract

There is a long-standing debate as to whether recollection is a continuous/graded process or a threshold/all-or-none process. In the current spatial memory functional magnetic resonance imaging (fMRI) study, we examined the hippocampal activity distributions—the magnitude of activity as a function of memory strength—to determine the nature of processing in this region. During encoding, participants viewed abstract shapes in the left or right visual field. During retrieval, old shapes were presented at fixation and participants classified each shape as previously in the “left” or “right” visual field followed by an “unsure”–“sure”–“very sure” confidence rating. The contrast of left-hits and left-misses produced two activations in the hippocampus. The hippocampal activity distributions for left shapes and right shapes were completely overlapping. Critically, the magnitude of activity associated with right-miss-very sure responses was significantly greater than zero. These results support the continuous model of recollection, which predicts overlapping activity distributions, and contradict the threshold model of recollection, which predicts a threshold above which only one distribution exists. Receiver operating characteristic analysis did not distinguish between models. The present results demonstrate that the hippocampus operates in a continuous manner during recollection and highlight the utility of analyzing activity distributions to determine the nature of neural processing.

## 1. Introduction

Long-term memory can be based on non-detailed familiarity or detailed recollection. Familiarity is widely believed to be a continuous process, ranging in strength from weak to intermediate to strong. However, the nature of recollection has been a topic of debate. Until about a decade ago, recollection was widely thought to be an all-or-none threshold process, where memories are either completely remembered or forgotten [1,2,3]. However, a growing body of recent behavioral evidence indicates that recollection is a continuous process [4,5,6].

**Figure 1:**
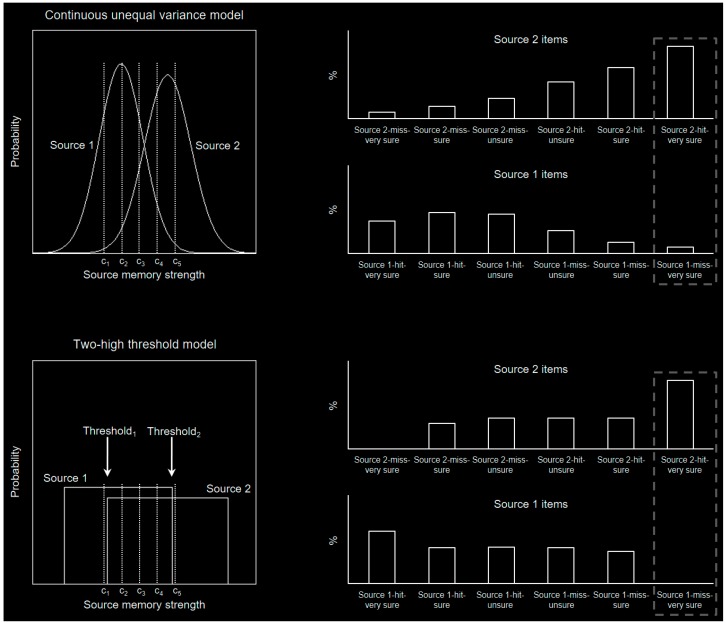
Models of recollection and event distributions. **Top**, continuous unequal variance model and corresponding percentage for each event type. **Bottom**, two-high threshold model and corresponding percentage for each event type.

The two models of recollection are formally referred to as the continuous unequal variance model and the two-high threshold model [7]. Figure 1, left, illustrates both of these models during memory for one of two sources/contexts. For example, during encoding, items could be presented in green (source 1) or red (source 2). During retrieval, the same items could be presented at fixation in gray and participants would make a confidence rating ranging from “very sure green” to “very sure red”. Each confidence rating depends on an item’s source memory strength and criteria placement (in this illustration, C_1_, C_2_, C_3_, C_4_, and C_5_). Memory strength greater than C_5_ would yield a “very sure source 2” response, memory strength between C_4_ and C_5_ would yield a “sure source 2” response, memory strength between C_3_ and C_4_ would yield an “unsure source 2” response, memory strength between C_2_ and C_3_ would yield an “unsure source 1” response, and so on. The continuous unequal variance model dictates that the sources have Gaussian distributions of memory strength that can have unequal variance (Figure 1, top left). The two-high threshold model dictates that there are two thresholds (threshold_1_ and threshold_2_) beyond which only one source distribution exists (Figure 1, bottom left). Figure 1, right, shows the percentage associated with each event type generated from each model to the left (e.g., each rightmost bar is the area under the corresponding distribution to the right of C_5_). Correct and incorrect source memory responses are referred to as hits and misses, respectively. The continuous unequal variance model predicts that the event distributions (i.e., source 1 and source 2 hits and misses; Figure 1, right) will be completely overlapping (i.e., all magnitudes will be greater than zero), whereas the two-high threshold model predicts that there is a threshold above which high confidence hits but not high confidence misses will have magnitudes greater than zero (key differential predictions are illustrated within the dashed boxes).

The nature of processing in the hippocampus is of importance as this region is known to be associated with recollection [6,8]. One study assessed whether the hippocampus operated in a continuous manner or a threshold manner by evaluating the activity distributions from this region during spatial memory [9]. Abstract shapes were shown to the left or right of fixation during encoding. During retrieval, old and new shapes were presented at fixation and participants classified each shape as “old-left”, “old-right”, or “new”, followed by an “unsure”–“sure” confidence rating. The contrast of old-left-hits and old-left-misses produced an increase in activity within one region of the hippocampus. The magnitude of activity associated with high confidence misses (old-right-miss-sure responses) was not significantly greater than zero, which was taken to support the threshold model. Thus, although previous behavioral results have supported the continuous model of recollection, previous hippocampal results have suggested that this region operates in a threshold manner during recollection [6]. However, in the previous study that evaluated hippocampal activity distributions [9], the activity associated with old-right-miss-sure responses was positive in magnitude and the standard error was large (see Section 4). As compared to that study, the current study was designed to have relatively smaller standard errors. First, we increased the number of participants from 12 to 16. Second, we increased the number of confidence ratings from two to three (“unsure”–“sure”–“very sure”). The “sure” response in the previous study can be assumed to reflect a mixture of cognitive processes, which would increase the standard error associated with this event type. In the current study, the three confidence ratings can be assumed to reflect more isolated cognitive processes and result in smaller standard errors. It is imperative to understand the operating model of the hippocampus, as this provides insight into the type of processing conducted by this region.

In the current spatial memory functional magnetic resonance imaging (fMRI) study, we analyzed the source memory distributions generated from behavioral responses and hippocampal activity to assess whether recollection operated in a continuous manner or a threshold manner. To anticipate the results, the behavioral response distributions and hippocampal activity distributions supported the continuous model of recollection and contradicted the threshold model of recollection.

## 2. Materials and Methods

### 2.1. Participants

Sixteen participants from the Boston College community completed the study (13 females, age range 22–28 years). Participants were right-handed, had normal or corrected-to-normal vision, were between the ages of 18–35, were native English speakers, were not pregnant, and had no metal in their bodies. Each participant was compensated $10 for the behavioral training session and $25 per hour (approximately $100) for the fMRI session. The Boston College Institutional Review Board approved the protocol (identification code: 10.008, initial approval date: 9 December 2009). Informed and written consent was obtained prior to the behavioral training session.

### 2.2. Stimulus Protocol

Participants completed a one-quarter length run and a full-length run during the behavioral training session and seven to eight full-length runs during the fMRI session. During fMRI, one participant completed seven runs due to time limitations. The remaining participants completed eight runs; however, for one participant, a stimulus protocol was accidentally repeated and the repeated run was discarded.

**Figure 2:**
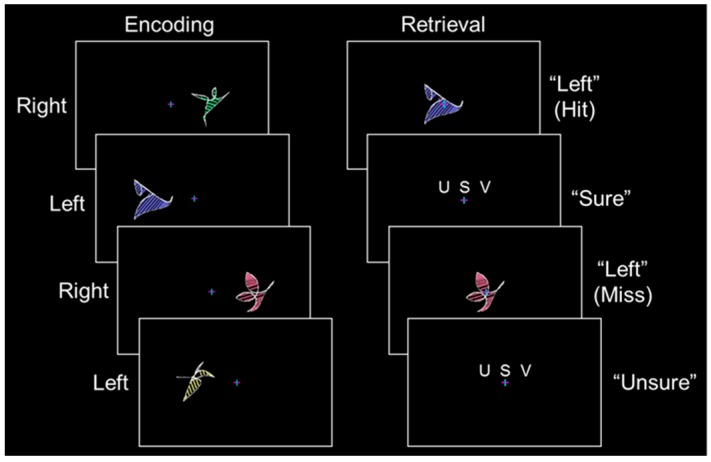
Experimental paradigm. **Left**, during encoding, participants viewed abstract shapes to the left or right of fixation (item types are shown to the left). **Right**, during retrieval, old items from encoding were presented at fixation and participants classified each shape as previously on the “left” or “right” followed by an “unsure”–“sure”–“very sure” confidence rating (possible responses and corresponding event types are shown to the right).

During the encoding phase of each full-length run, 32 abstract shapes (half in the left visual field and half in the right visual field) spanning 6.7° of visual angle were presented with their nearest edge 3.6° of visual angle from a central fixation cross (Figure 2, left). The shapes were designed to minimize visual encoding strategies (for information on shape construction, see Slotnick and Schacter [10]). Each shape was displayed for 2.5 s followed by a 0.5 s fixation period. Shape sets were presented three times, with each shape set randomized and presented sequentially. Participants were instructed to remember each shape and its spatial location.

Before each retrieval phase, an instruction screen was displayed for 8 s followed by a 2 s fixation period. During the retrieval phase of each full-length run, the 32 (old) shapes from encoding were randomized and each shape was presented at fixation for 3.0 s followed by a confidence rating reminder screen for 2.5 s and a fixation period of 0.5 to 4.5 s (Figure 2, right). This resulted in an inter-trial-interval of 6.0 to 10.0 s, which is sufficient to allow for the deconvolution of the hemodynamic response. Although the previous study that evaluated hippocampal activity distributions presented new shapes during retrieval [9], only old shapes were employed in the present study to increase the number of these critical event types. Although the lack of new items in the present study could affect criteria placement, this would not affect the distribution shapes and the predictions of each model (see Figure 1). Participants pressed response buttons with their left hand to classify each shape as previously presented in the “left” or “right” visual field followed by an “unsure”–“sure”–“very sure” confidence rating. In the previous study that evaluated hippocampal activity distributions “unsure” confidence ratings could correspond to forgotten old items or new items [9], while in the present study “unsure” confidence ratings could only correspond to forgotten old items. Of importance, this difference was not of importance as the key analyses were only conducted with confident responses.

For both the encoding phase and the retrieval phase, no more than three shapes of a given type were sequentially presented and participants were instructed to maintain fixation. Shapes were never repeated across runs and shape location (i.e., left and right) was counterbalanced across participants using a Latin square design.

### 2.3. Data Acquisition and Pre-processing

A Siemens 3 Tesla Trio Scanner (Siemens, Erlangen, Germany) with a 32-channel head coil was used to acquire imaging data. Functional images were acquired with an echo planar imaging sequence (repetition time (TR) = 2000 ms, echo time (TE) = 30 ms, flip angle = 90°, field-of-view = 256 × 256 mm^2^, acquisition matrix = 64 × 64, slices = 33, slice acquisition order = interleaved bottom-to-top, slice thickness = 4 mm, no gap; 4 mm isotropic resolution). Anatomic images were acquired with a magnetization prepared rapid gradient echo sequence (TR = 30 ms, TE = 3.3 ms, flip angle = 40°, field-of-view = 256 × 256 mm^2^, acquisition matrix = 256 × 256, slices = 128, slice thickness = 1 mm; 1.33 × 1 × 1 mm resolution). Analyses were conducted with BrainVoyager 20.0 (Brain Innovation B.V., Maastricht, The Netherlands). Functional pre-processing included slice-time correction, motion correction, and removal of temporal components below two cycles per run length (using a general linear model to remove low frequency Fourier basis sets). Voxels were resampled at 3 mm^3^. To maximize spatial resolution, spatial smoothing was not conducted. Anatomic and functional images were transformed into Talairach space.

### 2.4. General Linear Model Analysis

A random-effect general linear model analysis was conducted. Each event type was modeled based on its onset and the subsequent behavioral response (if a response was made). Encoding trials and no-response trials were assumed to have durations of 2.5 s and the mean level of activity for each run was modeled with a constant. The contrast of correct spatial location memory (left-hits) and incorrect spatial location memory (left-misses; i.e., “left”/left > “right”/left) was used to isolate activity associated with spatial memory for shapes in the left visual field and the analogous contrast (i.e., “right”/right > “left”/right) was used to isolate activity associated with spatial memory for shapes in the right visual field. The previous study that evaluated hippocampal activity distributions isolated hippocampal activity using the conjunction of left-hits > left-misses and left-hits > right-hits in addition to the analogous conjunction for right visual field stimuli [9]. However, these relatively conservative conjunctions did not produce any significant hippocampal activity using the present data; therefore, we used the standard hit > miss contrast in each visual field in an effort to identify multiple hippocampal activations. For all contrasts, an individual voxel threshold of *p* < 0.001 was enforced, false discovery rate corrected for multiple comparisons to *p* < 0.05. False discovery rate correction for multiple comparisons does not require a minimal cluster extent but rather, for a given individual voxel threshold, ensures an acceptable rate of false positives across the entire brain [11]. Known anatomical distinctions within the medial temporal lobe were used to localize hippocampal activations [12,13,14,15]. Activations were localized on the group average anatomic volume. Each Talairach coordinate refers to the voxel with peak activity.

### 2.5. Hippocampal Activity Distribution Analysis

For each hippocampal activation, event-related magnitudes were extracted from active voxels within a 5 mm cube (centered on the activation) from −2 to 12 s after stimulus onset (baseline corrected from −2 to 0 s). To ensure activation magnitudes were greater than, or equal to, baseline, which corresponds to a lower boundary on neural firing at zero spikes per second, the minimum activation magnitude across all event types was subtracted from each activation timecourse [9]. As fMRI activity can be assumed to reflect the underlying neural activity [16], we subtracted the minimum activation magnitude in an effort to make the zero point in the magnitude of fMRI activity correspond to the zero point of neural activity. This zero point in the magnitude of fMRI activity is analogous to no responses in behavior. To ensure the hippocampal activity distribution results did not depend on baseline correction, we also compared the magnitude of activity associated with miss-sure responses and miss-very sure responses as both of these trial types have the same baseline (which were subtracted out in the comparison). The threshold model predicts that the magnitude of activity associated with miss-sure responses will be significantly greater than the magnitude of activity associated with miss-very sure responses (see Figure 1). For each event type, the mean magnitude of activity from 4 to 6 s after stimulus onset was used for analysis (i.e., the expected maximum amplitude of the hemodynamic response). Hippocampal activity distributions were generated by plotting the magnitude of activity as a function of memory strength where, from left to right, responses were “right-very sure”, “right-sure”, “right-unsure”, “left-unsure”, “left-sure”, and “left-very sure”. This corresponded to the following event types (mean trial numbers are shown in parenthesis) for left shapes: left-miss-very sure (3.1), left-miss-sure (9.5), left-miss-unsure (18.1), left-hit-unsure (21.3), left-hit-sure (31.1), left-hit-very sure (42.6), and the following event types for right shapes: right-hit-very sure (42.9), right-hit-sure (33.6), right-hit-unsure (22.2), right-miss-unsure (14.8), right-miss-sure (10.6), right-miss-very sure (1.6). The analysis was conducted after excluding three participants who made no right-miss-very sure responses. Although the numbers of trials associated with high confidence misses were relatively low, this would be expected to increase the corresponding standard errors and produce null results. Since significant results were observed (see Section 3.2), this was not of concern. As the direction of the statistical tests were known a priori, given that only an increase in the magnitude of activity relative to baseline reflects a memory-related activation, one tailed *t*-tests were employed. The behavioral response distribution analysis mirrored the hippocampal activity distribution analysis, except for the comparison between miss-sure responses and miss-very sure responses conducted as a test that was independent of baseline.

### 2.6. ROC Analysis

To compute activation percentages as a function of memory strength, each left shape activation magnitude was divided by the sum of all left shape activation magnitudes, and each right shape activation magnitude was divided by the sum of all right shape activation magnitudes. Hit rates were then computed by cumulating the probabilities from the highest to lowest memory strength for that stimulus type (e.g., left), and false alarm rates were computed by cumulating the probabilities from lowest to highest memory strength for the other stimulus type (e.g., right). Each hippocampal ROC was generated by plotting these hit rates versus false alarm rates. The behavioral ROC was generated by plotting the hit rates versus false alarm rates based on the percentage of responses as a function of memory strength for each event type (left items and right items were arbitrary defined as source 1 and source 2, respectively; see Figure 1). For hippocampal ROC analysis we have assumed that the parametric estimates of neuronal activity are analogous to the parametric estimates of behavioral responses. That is, behavioral ROC analysis is based on the number of responses associated with each event type, as is commonly done, while hippocampal ROC analysis is based on the magnitude of activity associated with each event type. Hippocampal ROC results are only valid if this assumption is correct. The two-high threshold recollection model (with parameters *R*_1_ and *R*_2_) and the continuous unequal variance model (with parameters *d*’ and σ_s2_/σ_s1_, the ratio of source distribution standard deviations) were fit to each ROC by adjusting model parameters using maximum likelihood estimation. The log-likelihood chi-square value was used to assess the adequacy of each model, where a lower chi-square value reflects a better fit (*p* > 0.05 indicates an adequate fit).

## 3. Results

### 3.1. Behavioral Results

**Figure 3:**
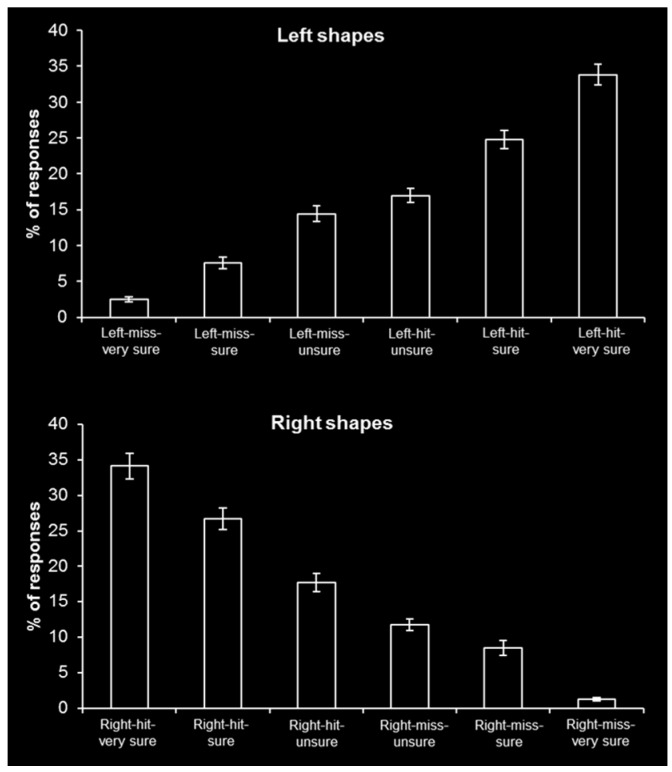
Behavioral response distributions. Percentage of responses for each left shape and right shape event type (mean ± S.E.).

Figure 3 shows the behavioral response distributions (in percentage of responses) for each left shape and right shape event type. Of importance, the percentage of highest confidence misses were both significantly greater than zero (left-miss-very sure, *t*(15) = 6.61, *p* < 0.001; right-miss-very sure, *t*(15) = 4.95, *p* < 0.001). These behavioral findings support the continuous unequal variance model of recollection and contradict the two-high threshold model of recollection.

**Figure A1:**
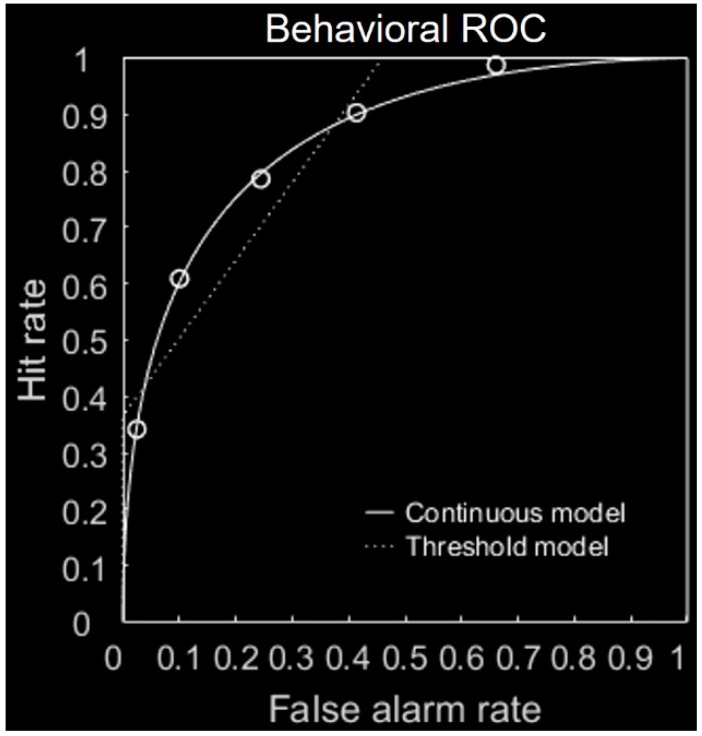
Behavioral ROC (circles) with the best-fit continuous unequal variance model and two-high threshold model ROCs (key at the bottom right).

A chi-square analysis revealed that the behavioral ROC was not adequately fit by either the continuous unequal variance model (χ^2^(3) = 30.14, *p* < 0.001) or the two-high threshold model (χ^2^(3) = 525.40, *p* < 0.001; Figure A1). It is notable that when forgotten items are included in the analysis, this can artifactually flatten the ROC and result in an inadequate fit for the continuous model [6,7]. Recollection-based ROCs that do not include forgotten items in the analysis are adequately fit by the continuous model but not the threshold model [7,17,18]. As a significant proportion of “unsure” responses can be assumed to reflect forgotten items, the present behavioral ROC results are not inconsistent with the continuous model. Still, as neither model adequately fit the behavioral ROC, the chi-square analysis results did not distinguish between the continuous model of recollection or the threshold model of recollection.

### 3.2. Hippocampal Results

**Figure 4:**
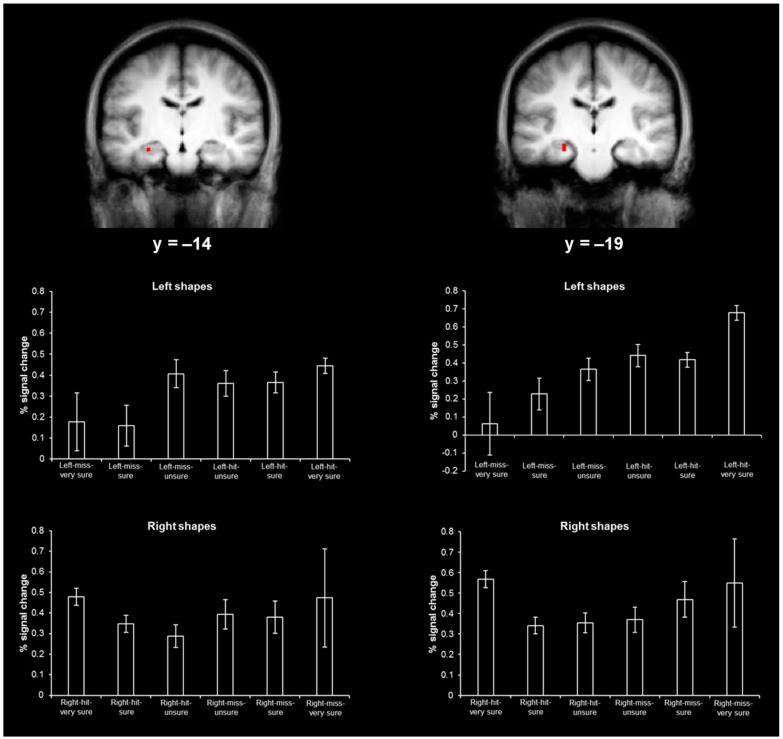
Hippocampal activity associated with spatial memory and hippocampal activity distributions. **Top**, hippocampal activity associated with left-hits versus left-misses (in red; coronal views). **Bottom**, hippocampal activity distributions (percent signal change for each event type) for left-shapes and right shapes corresponding to each hippocampal activation above.

The contrast of left-hits and left-misses produced two activations in the hippocampus (Figure 4, top; *x* = −27, *y* = −14, *z* = −15, size = 54 mm^3^; *x* = −24, *y* = −19, *z* = −11, size = 27 mm^3^). The contrast of right-hits and right-misses did not produce any activity in the hippocampus, even at a reduced threshold of *p* < 0.01, uncorrected. This preferential hippocampal activity during memory for items in the left visual field has been observed in previous fMRI studies [9,19].

Figure 4, bottom, shows the hippocampal activity distributions (in percent signal change) for left shapes and right shapes. Of importance, for both activations, the magnitude of activity associated with the highest confidence misses (right-miss-very sure responses) was significantly greater than zero (bottom left, *t*(12) = 1.98, *p* < 0.05; bottom right, *t*(12) = 2.54, *p* < 0.05). It should be highlighted that left-miss-very sure responses were not expected to have magnitudes that were significantly greater than zero in these regions because they were identified by contrasting left-hits and left-misses (i.e., left-miss was the baseline event and was expected to have a relatively low magnitude of activity). In addition, for both activations, the magnitude of activity associated with right-miss-sure responses was not significantly greater than the magnitude of activity associated with right-miss-very sure responses (*ts*(12) < 1). These hippocampal findings support the continuous model of recollection and contradict the threshold model of recollection.

**Figure A2:**
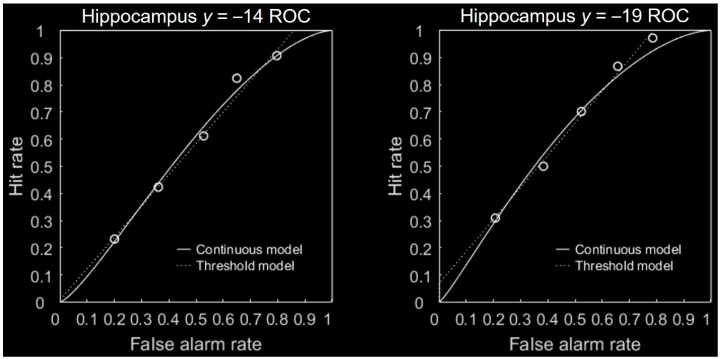
Hippocampal ROCs (circles) with the best-fit continuous unequal variance model and two-high threshold model ROCs (key at the bottom right of each ROC).

A chi-square analysis revealed that both hippocampal ROCs were adequately fit by the continuous unequal variance model (*y* = −14 region, χ^2^(3) = 4.67, *p* = 0.20; *y* = −19 region, χ^2^(3) = 5.73, *p* = 0.13) and the two-high threshold model (*y* = −14 region, χ^2^(3) = 4.00, *p* > 0.20; *y* = −19 region, χ^2^(3) = 3.91, *p* > 0.20; Figure A2). The adequate fit for both models is likely due to the relatively low signal strength in both regions (*y* = −14 region, *d*’ = 0.23; *y* = −19 region, *d*’ = 0.38), which corresponds to an ROC that lies close to the diagonal (i.e., the chance line) and should be well fit by both models. As both models adequately fit the hippocampal ROCs, the chi-square analysis results did not distinguish between the continuous model of recollection and the threshold model of recollection.

## 4. Discussion and Conclusions

In the present behavioral response distribution and the hippocampal activity distributions, there was no threshold above which only one source distribution existed. Specifically, in the behavioral response distribution, the percentage of left-miss-very sure and right-miss-very sure responses were significantly greater than zero. In the hippocampal activity distributions, the magnitude of activity associated with right-miss-very sure responses was significantly greater than zero. These findings support the continuous model of recollection and contradict the threshold model of recollection.

Although analysis of the behavioral response distributions and hippocampal activity distributions distinguished between the continuous model and the threshold model of recollection, the ROC analysis did not distinguish between these models. The behavioral ROC was not adequately fit by either model and the hippocampal ROCs were adequately fit by both models. This indicates that behavioral and hippocampal distribution analysis is a more sensitive measure than ROC analysis in distinguishing between the continuous model of recollection and the threshold model of recollection. This is likely because distribution analysis focuses on the differential prediction of the single critical event type (i.e., whether or not the magnitude of high confidence misses is significantly greater than zero). By contrast, the ROC is generated from all the event types, which could mask differential effects that exist. As mentioned previously, the hippocampal ROC analysis is based on the assumption that the parametric estimates of neural activity are analogous to the parametric estimates of behavioral responses and the hippocampal ROC results are only valid if this assumption is correct. This assumption is not critical to the present results as the chi-square analysis did not distinguish between the models of recollection.

**Figure A3:**
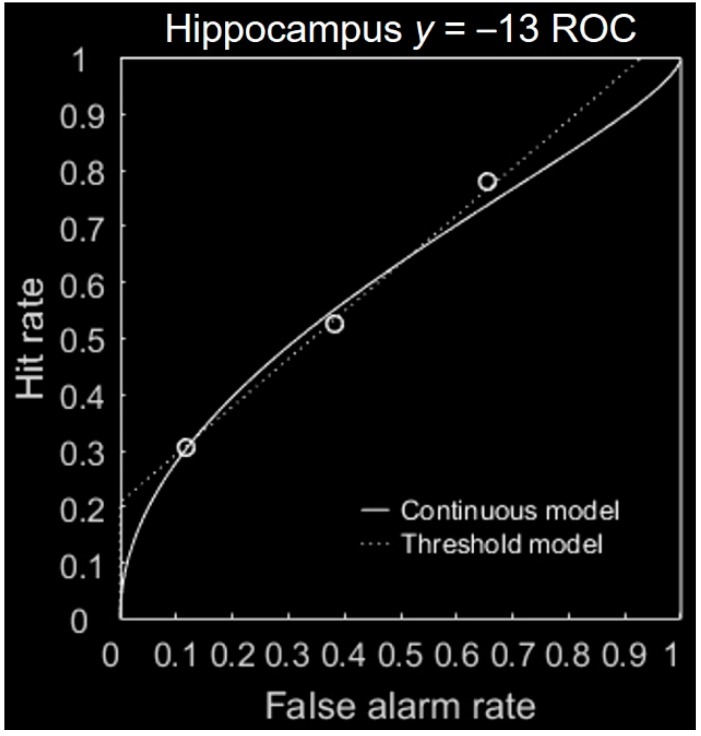
Hippocampal ROC from Slotnick and Thakral (2013) with the best-fit continuous unequal variance model and two-high threshold model ROCs (key at the bottom right).

As in the present study, Slotnick and Thakral analyzed the spatial memory hippocampal activity distribution to distinguish between the continuous model of recollection and the threshold model of recollection [9]. As mentioned in the Introduction, in that study, the conjunction of left-hits > left-misses and left-hits > right-hits produced one activation in the hippocampus and the magnitude of activity associated with old-right-miss-sure responses was not significantly greater than zero. However, the old-right-miss-sure activity was positive in magnitude (0.16% signal change) and the standard error was large (0.16). Therefore, this null finding can be attributed to the large standard error rather than old-right-miss-sure activity being zero in magnitude. The current study was designed to reduce this large standard error by increasing the number of participants and requiring three confidence response options. These modifications can explain why the present results were significant, which should be favored over the null finding of Slotnick and Thakral [9]. Slotnick and Thakral also reported that the threshold model but not the continuous model adequately fit the hippocampal activity ROC [9]. However, an equal variance continuous model was fit to the ROC, which assumes the variance for old-left items and old-right items are identical. Recent evidence indicates that the hippocampus is associated with memory for items previously presented in the left visual field to a greater degree than memory for items previously presented in the right visual field [18], which would be expected to produce unequal variances for old-left items and old-right items. As such, the unequal variance continuous model should have been fit to the hippocampal ROC. We fit the continuous unequal variance model and the two-high threshold model to the hippocampal ROC from Slotnick and Thakral [9] and a chi-square analysis revealed that both models provided an adequate fit (continuous unequal variance model, χ^2^(1) = 2.89, *p* = 0.089; two-high threshold model, χ^2^(1) < 1; Figure A3). Thus, as in the current study, ROC analysis did not distinguish between the continuous model of recollection and the threshold model of recollection.

The present behavioral response distribution and hippocampal activity distributions suggest that recollection is a continuous process. These findings support recent behavioral evidence that recollection is a continuous process [4,5,6]. Our findings further suggest that continuous processing in the hippocampus contributes to continuous behavioral processing. Future work will be needed to evaluate the nature of processing in other neural regions to determine how processing across the brain gives rise to continuous behavioral processing during recollection.

The current findings indicate that the hippocampus operates in a continuous manner during recollection. This has implications for other lines of memory research. For instance, computational models of hippocampal function should not assume that this region operates in a threshold/all-or-none manner [20]. In addition, prospective memory (i.e., imagining the future) relies on episodic memory in which specific details from past events are recalled and recombined to form imagined scenarios, and prospective memory has been associated with activity in the hippocampus [21,22]. The present results indicate that during prospective memory, hippocampal activity does not reflect retrieval of details in a threshold/all-or-none manner, but rather reflects retrieval of graded details, ranging in strength from weak to intermediate to strong. While the current hippocampal activity distributions support the continuous model of recollection, future research could manipulate experimental factors such as stimulus type, number of repetitions, and encoding-retrieval delay to evaluate hippocampal activity distributions under different conditions. It is predicted that these results will support the present findings that the hippocampus operates in a continuous manner during recollection.
